# Preference of methadone maintenance patients for the integrative and decentralized service delivery models in Vietnam

**DOI:** 10.1186/s12954-015-0063-0

**Published:** 2015-09-17

**Authors:** Bach Xuan Tran, Long Hoang Nguyen, Huong Thu Thi Phan, Linh Khanh Nguyen, Carl A. Latkin

**Affiliations:** Institute for Preventive Medicine and Public Health, Hanoi Medical University, Hanoi, Vietnam; Johns Hopkins Bloomberg School of Public Health, Baltimore, MD USA; School of Medicine and Pharmacy, Vietnam National University, Hanoi, Vietnam; Authority of HIV/AIDS Control, Ministry of Health, Hanoi, Vietnam; Illinois Wesleyan University, Bloomington, USA

**Keywords:** Methadone, Integrative, Decentralized, Preference, MMT, Vietnam

## Abstract

**Background:**

Integrating and decentralizing services are essential to increase the accessibility and provide comprehensive care for methadone patients. Moreover, they assure the sustainability of a HIV/AIDS prevention program by reducing the implementation cost. This study aimed to measure the preference of patients enrolling in a MMT program for integrated and decentralized MMT clinics and then further examine related factors.

**Methods:**

A cross-sectional study was conducted among 510 patients receiving methadone at 3 clinics in Hanoi. Structured questionnaires were used to collect data about the preference for integrated and decentralized MMT services. Covariates including socio-economic status; health-related quality of life (using EQ-5D-5 L instrument) and HIV status; history of drug use along with MMT treatment; and exposure to the discrimination within family and community were also investigated. Multivariate logistic regression with polynomial fractions was used to identify the determinants of preference for integrative and decentralized models.

**Results:**

Of 510 patients enrolled, 66.7 and 60.8 % preferred integrated and decentralized models, respectively. The main reason for preferring the integrative model was the convenience of use of various services (53.2 %), while more privacy (43.5 %) was the primary reason to select stand-alone model. People preferred the decentralized model primarily because of travel cost reduction (95.0 %), while the main reason for not selecting the model was increased privacy (7.7 %). After adjusting for covariates, factors influencing the preference for integrative model were poor socioeconomic status, anxiety/depression, history of drug rehabilitation, and ever disclosed health status; while exposure to community discrimination inversely associated with this preference. In addition, people who were self-employed, had a longer duration of MMT, and use current MMT with comprehensive HIV services were less likely to select decentralized model.

**Conclusion:**

In conclusion, the study confirmed the high preference of MMT patients for the integrative and decentralized MMT service delivery models. The convenience of healthcare services utilization and reduction of geographical barriers were the main reasons to use those models within drug use populations in Vietnam. Countering community stigma and encouraging communication between patients and their societies needed to be considered when implementing those models.

## Introduction

Illicit drug abuse is a global public health issue and a major risk factor for spreading HIV/AIDS, especially in low-and middle-income Asian countries [1]. Previous evidences suggested that using illicit drug significantly reduces antiretroviral treatment (ART) access, adherence to ART, and viral resistance in people living with HIV [2–4]. In such cases, opioid substitution treatment for drug users plays an indispensable role in HIV/AIDS prevention strategies [5].

Methadone maintenance treatment (MMT) has been widely used to treat opioid dependence [5, 6]. World Health Organization (WHO) considers methadone as a priority drug for opioid management [7]. Prior reviews showed that MMT minimizes opioid use, crime activities, HIV-related risk behaviors and diseases, as well as facilitated HIV/AIDS care services access and improved quality of life [6, 8]. Thus, implementing and scaling-up MMT program has been considered a cost-effective intervention in both developed and developing countries [9–11].

Opioid dependence treatment is recommended to be integrated into comprehensive services, including HIV voluntary counseling and testing, antiretroviral treatment, and primary health care [12]. This comprehensive model is effective as illicit drug users are at high risks not only for acquiring HIV/AIDS but also for other physical and mental health problems [13–16]. Integrative delivery model has been demonstrated to promote not only positive health outcomes but also increased ART and medical care adherence [17–20]. MMT integration into other health care services and decentralization to primary healthcare facilities may also increase the accessibility for drug users. A recent survey suggested that decentralizing MMT service plays an important role in reducing travel distance, which is a major barrier among people who inject drugs (PWID) [21]. Providing all services within a single site could also ensure the sustainability of HIV/AIDS programs by reducing operational cost [9, 22–25], as well as alter the paradigm to deliver services, from vertical (only MMT facilities provide MMT services) to diagonal (integrative model) [26].

The HIV epidemic in Vietnam is recognized in a concentrated stage, which is primarily driven by PWIDs [13, 8]. To prevent HIV transmission in this high-risk population, MMT is selected to be the primary substitution opioid therapy in national HIV/AIDS preventive strategies [27–31]. Current data reports that 31,162 drug users have currently participated in MMT program nationwide [32], accounting for approximately 17 % of drug users managed (181,000) in Vietnam [33]. With strong political will and commitment, the Vietnam government aims to scale-up MMT services to cover 80,000 drug users in 2015 [11]. However, the reduction of financial support from foreign donors (Global Fund up to 2017 and PEPFAR up to 2018) in the next few years is a key barrier to achieving this goal [32]. Integrating MMT services into other health care settings and decentralizing MMT into primary health care can be used as an alternative pathway to deliver MMT services to large drug user population with low cost and high efficiency.

Measuring the preference of patients for different MMT service delivery models is essential to evaluate the feasibility of implementing those models. However, presently, no literature has been published on this topic in Vietnam and worldwide. Therefore, this study aimed to measure the preference of patients enrolling in MMT programs for integrated and decentralized MMT clinic and then examine the related-factors of those preferences.

## Methods

### Study setting, sample size, and sampling method

A cross-sectional study was performed from June to August 2013 in Hanoi, a Vietnamese epicenter of drug user. The eligible criteria for selecting MMT clinics included the following: (1) providing MMT services; (2) consisting of different administrative levels; (3) having at least 100 MMT patients in each clinic for study. In 2013, there were six MMT clinics available in Hanoi. We prepared the list of those clinics and randomly selected three clinics among those meeting eligible criteria, including Tu Liem, Ha Dong, and Long Bien District Health Centers (DHC). Along with MMT service, MMT clinics at Tu Liem and Long Bien DHC provide antiretroviral treatment (ART), voluntary HIV testing and counseling (VCT), and general health care (GH), while Ha Dong is a polyregional clinic which provides MMT and general health care services. Table 1 lists the characteristics of study settings.

**Table 1:** Study settings and sample size

Settings	Site name	Type of services	Patient load	Sample size
Tu Liem District	District Health Centre	MMT + VCT + ART + GH	300	200
Long Bien District	District Health Centre	MMT + VCT + ART + GH	200	100
Ha Dong District	Regional Polyclinic	MMT + GH	300	210

All MMT patients at selected clinics were clearly explained about the purposes and invited to participate in the study following the eligible criteria: (1) were 18 years old or above; (2) attended clinics during study period; (3) agreed to participate in the study. A total of 510 patients agreed to enroll in this study, accounting for approximately 80 % of patients in those clinics.

### Measures and instruments

Study subjects were invited to interview in a designated room to ensure the confidentiality where, they were provided the information about the purposes and objectives of this study; and a written informed consent was signed for patients who agreed to participate. Data was collected using structured questionnaire by well-trained surveyors, including master students and experts in HIV/AIDS-related fields.

In order to investigate the preference for different delivery models, several following steps were implemented. First, the surveyors described the current stand-alone and integrated MMT delivery models for patients and asked for their preference for each clinic model. Because they still used services in integrated clinics, they were easy to imagine this model. However, there was none of the stand-alone model in Hanoi, therefore, interviewers had to describe the characteristics of stand-alone clinics in other Vietnamese provinces and show images in order to help patients to understand this kind of model. In addition, patients were asked if they preferred receiving MMT at decentralized models that were offered at their commune health stations. Patients were also mentioned that if these models were available in their areas, they would have equal opportunities to access without any barrier.

For each selection, interviewers also asked about the reason of preference. The list of reasons for selection was developed based on the advantages of those models, which were by reviewing previous literatures, including: close to home, fewer visits to different services, convenience in multiple services use [21, 34, 35], health workers had more aware of patients’ status, better quality of care [17–20, 36], more privacy, and less discrimination [37].

Based on those literatures, we also developed a conceptual framework in order to determine associated factors for the preference. Additional measures included: socio-economic (gender, education attainment, marital status, religion and employment, income), health, and HIV status. Income per capita was used to categorize patients into five quintile groups (from poorest to richest). HIV status was collected through self-report from MMT patients and checked with health staffs in MMT clinics. Health status was measured using EQ-5D-5 L instrument, which considers five dimensions (mobility, self-care, usual activities, pain/discomfort, and anxiety/depression) with five levels of response [38]. Furthermore, data on history of drug injection, current drug use, age of initial drug use, history of drug rehabilitation, and duration of MMT treatment were also collected. Finally, experiences in family and community discrimination as well as ever disclosed health status, including drug addiction and other related health problems were investigated.

### Statistical analysis

STATA software version 12.0 (StataCorp. LP, College Station, USA) was used to analyze the data. *T*-test and *χ*^2^ test were used to show the difference of preference for service delivery models among characteristics of interest. To identify the associated factors with the preference for integrative and decentralized models, multivariate binominal logistic regression, combining with fractional polynomials models for duration of MMT treatment (by month), was performed to determine non-linear associations. Odd ratios (ORs) from regression models were displayed with 95 % CI. Backward stepwise selection strategy was utilized to remove non-significant factors, with *p* values of log-likelihood ratio test <0.1 and was the threshold to include variables. The statistical significant was determined with a *p*-value <0.05.

### Ethics approval

The study was approved by The Ethical Committee of Authority for HIV and AIDS Control at the Vietnamese Ministry of Health.

## Results

Table 2 describes the socio-economic status of respondents. A total of 510 patients participated in this study; 98.4 % were male and 53.7 % had less than high school education. Seventy percent of subjects were living with their spouse and 92.7 % have a cult of ancestors. Most respondents were self-employed (52.7 %) or unemployed (26.6 %).

**Table 2:** Socioeconomic characteristics of MMT patients

	MMT + general health care		MMT + comprehensive HIV services		Total		*p* value
	*N*	%	*N*	%	*N*	%	
Male	206	98.1	293	98.7	499	98.4	0.62
Education							
Illiterate	4	1.9	7	2.4	11	2.2	0.41
Elementary	27	12.9	34	11.5	61	12.0	
Secondary	86	41.0	114	38.4	200	39.5	
High	81	38.6	118	39.7	199	39.3	
Vocational	7	3.3	6	2.0	13	2.6	
University	5	2.4	18	6.1	23	4.5	
Marital status							
Single	47	22.4	59	19.9	106	20.9	0.76
Live with spouse	147	70.0	208	70.0	355	70.0	
Live with partner	1	0.5	1	0.3	2	0.4	
Divorced	15	7.1	28	9.4	43	8.5	
Widow	0	0.0	1	0.3	1	0.2	
Religion							
Cult of ancestors	198	94.3	272	91.6	470	92.7	0.35
Buddhism	10	4.8	17	5.7	27	5.3	
Catholic	2	1.0	4	1.4	6	1.2	
Protestant	0	0.0	4	1.4	4	0.8	
Employment							
Unemployed	53	25.2	82	27.6	135	26.6	0.32
Self-employed	112	53.3	155	52.2	267	52.7	
White collars	5	2.4	10	3.4	15	3.0	
Workers, farmers	18	8.6	13	4.4	31	6.1	
Students	0	0.0	2	0.7	2	0.4	
Other jobs	22	10.5	35	11.8	57	11.2	

Self-report health status and perceived discrimination of MMT clients are shown in Table 3 regarding to the preference for MMT models, of which two third of patients preferred to use integrated service delivery. The minority of patients reported having problem of mobility, self-care, and usual activities (5.9, 3.8, and 3.9 %, respectively). The percentage of respondents suffering from pain/discomfort and anxiety/depression were 15.2 and 16.2 %, correspondingly, and people preferring integrated MMT models showed the significantly higher prevalence of these two factors than their counterparts. Most of patients reported HIV-negative status (86.4 %). In term of stigma, only 1.2 and 7.5 % of the patients were discriminated in their family and communities, respectively. Furthermore, 76.1 % of the patients reported disclosing their addiction and/or related health problems to at least on other person and this proportion was significantly higher among people preferring integrative models compared to their counterparts.

**Table 3:** Health status, perceived discrimination and history of drug addiction among MMT patients by different service preference

	Preference for service delivery model				All		
	Stand-alone		Integrative				*p* value
	*n*	%	*n*	%	*n*	%	
All patients	170	33.3	340	66.7	510	100.0	
Self-reported health problems							
Mobility	14	8.2	16	4.8	30	5.9	0.12
Self-care	8	4.7	11	3.3	19	3.8	0.42
Usual activities	6	3.5	14	4.2	20	3.9	0.73
Pain or discomfort	11	6.5	66	19.6	77	15.2	<0.0
Anxiety or depression	10	5.9	72	21.4	82	16.2	<0.01
Self-reported HIV status							
Negative	149	87.7	289	85.8	438	86.4	0.84
Positive	13	7.7	30	8.9	43	8.5	
N/A	8	4.7	18	5.3	26	5.1	
Discrimination							
Perceived in family	1	0.6	5	1.5	6	1.2	0.39
Perceived in community	18	10.6	20	5.9	38	7.5	0.06
Ever disclosed health status	117	68.8	271	79.7	388	76.1	0.01
Drug use							
History of drug injection	123	72.4	249	73.9	372	73.4	0.71
Concurrent drug use	6	3.5	9	2.7	15	3.0	0.59
	Mean	SD	Mean	SD	Mean	SD	
Age at first drug use	23.9	6.4	24.3	6.3	24.2	6.3	0.77
Time since first drug use (year)	13.6	6.2	13.3	6.1	13.4	6.1	0.31
Time since first drug inject(year)	10.4	5.1	9.9	5.2	10.1	5.2	0.20
Number of previous drug rehabilitation	4.7	5.6	4.7	6.1	4.7	5.9	0.47
MMT duration (month)	20.2	11.7	21.1	11.9	20.8	11.8	0.78

Drug use behaviors were also presented in Table 3. Among 510 patients, 73.4 % had experiences about injecting drug and 3.0 % of the patients reported currently using illicit drug. There was no statistical difference between people preferring stand-alone and integrated delivery models in age at first drug use, time since first drug use/injection, and time of prior drug rehabilitation. The mean of MMT duration among patients was 20.8 (SD = 11.8) months, with no difference between two groups.

The reasons for selecting different MMT service delivery models are presented in Table 4. The main reason that people preferred to utilize integrated model included more convenient to use other services (53.2 %), better quality in care (38.2 %), and need fewer visits for different services (29.5 %), while more privacy (43.5 %) and better care (22.9 %) were the primary reasons to select stand-alone model. In terms of decentralized MMT services at commune level, people preferred for this model mainly because of travel cost reduction (95.0 %), while the main reason for not selecting the model was to have more privacy (7.7 %). The results also showed no statistical difference for preferences regarding to current services (MMT + GH services versus MMT + comprehensive HIV services).

**Table 4:** Reason for integrative and decentralized services preference among MMT patients

	Preference for service delivery model				All		
	Stand-alone		Integrative				*p* value
	*n*	%	*n*	%	*n*	%	
All	170	33.3	340	66.7	510	100.0	
Stratified by current services							
MMT + general health	80	38.1	130	61.9	210	100.0	0.07
MMT + comprehensive HIV services	90	30.3	207	69.7	297	100.0	
Reasons							
Closer to home, reduced travel cost	12	7.1	72	21.2	84	16.5	<0.01
Fewer visits to different services	4	2.4	97	28.5	101	19.8	<0.01
More convenient in multiple services use	15	8.8	181	53.2	196	38.4	<0.01
Health workers are more aware of patients’ status	8	4.7	95	27.9	103	20.2	<0.01
Better health care quality	39	22.9	130	38.2	169	33.1	<0.01
More privacy	74	43.5	32	9.4	106	20.8	<0.01
Less discrimination	7	4.1	4	1.2	11	2.2	0.03
	Preference for decentralized MMT services at commune level						*p* value
	No		Yes		All		
	*n*	%	*n*	%	*n*	%	
All	194	39.2	301	60.8	510	100.0	
Stratified by current services							
(MMT + GH)	73	35.1	135	64.9	208	100.0	0.11
MMT + comprehensive HIV services	121	41.2	166	57.8	287	100.0	
Reasons							
Closer to home, reduced travel cost	1	0.5	286	95.0	287	58.0	<0.01
Fewer visits to different services	0	0.0	6	2.0	6	1.2	0.05
More convenient in multiple services use	0	0.0	16	5.3	16	3.2	<0.01
Health workers are more aware of patients’ status	0	0.0	0	0.0	0	0.0	-
Better health care quality	0	0.0	1	0.3	1	0.2	0.42
More privacy	15	7.7	2	0.7	17	3.4	<0.01
Less discrimination	4	2.1	0	0.0	4	0.8	0.01

Table 5 showed the results of reduced multivariate logistic regression with fraction polynomials. People were likely to select integrative MMT models if they had low income, reported anxiety/discomfort, previous drug rehabilitation experience, and ever disclosed health status. Meanwhile, being Catholic and facing community discrimination were associated with greater likelihood to select the stand-alone model. The findings in Table 5 also showed that patients belonging to low-income group and ever disclosed health status were more likely to prefer for decentralized MMT models, while respondents who were self-employed, receiving MMT treatment in comprehensive HIV service clinics, and longer duration of MMT were less likely to choose decentralized clinics.

**Table 5:** Factors associated with patients preferences for the integrative and decentralized MMT services

	Preference for integrative MMT models		Preference for decentralized MMT models	
	OR	95 % CI	OR	95 % CI
Religion (Ref—Cult of ancestors)				
Catholic	0.18	(0.03–1.09)		
Employment (Ref—Unemployed)				
Self-employed			0.55***	(0.36–0.84)
Current MMT services (Ref—MMT + GH)				
MMT + Comprehensive HIV services			0.59**	(0.38–0.91)
Income per head (Ref—Poorest)				
Poor	1.99**	(1.10–3.60)	1.65	(0.94–2.90)
Self-reported health problems				
Self-care vs. no			3.28	(0.70–15.28)
Anxiety/depression vs. no	3.50***	(1.67–7.34)		
Number of previous drug rehabilitation (Ref—none)				
1–5 times	2.35**	(1.22–4.53)		
6–10 times	2.02*	(0.94–4.34)		
Concurrent drug use vs. non-concurrent			1.46	(0.92–2.33)
Duration on MMT (months)	1.01	(0.99–1.02)	0.93**	(0.88–0.99)
Discrimination				
By family vs. none	5.83	(0.56–60.43)		
By community vs. none	0.40**	(0.18–0.88)		
Ever disclosed health status vs. not yet	1.89**	(1.15–3.10)	1.56	(0.94–2.59)

Significant with *p < 0.05; **p < 0.01; ***p < 0.000

## Discussion

Integrating MMT into other health care settings and decentralizing into primary health care facilities have a critical role in ensuring the sustainability of HIV/AIDS interventions in large drug-using populations. The findings of this study revealed the high prevalence of patients preferring integrated and decentralized models, with travel cost saving, convenience of using services, better care, and easy access for diverse health care services as primary reasons. The results also showed that income, mental health status, history of drug rehabilitation, and discrimination related with the preferences for integrative models, while occupation, current MMT models, and duration of MMT were associated with the preferences for decentralized models. This evidence contributes to inform the effective scaling up MMT program to provide comprehensive health care for drug use populations in Vietnam.

### Preference for integrative models

Empirical evidences about the benefits of integrative MMT service delivery models are mentioned in international literature. By combining different components of health care services into a single site; or providing referral between them, these models can address the unmet needs of drug users for medical services [39–41]. It could facilitate health care utilization and improve health outcome and treatment adherence among drug user population [36, 17–20]. Furthermore, patients can reduce their health care expenditure with timely access to primary care [34, 35, 21]. In this study, from patients’ perspective, convenience in using various health care services in a single site, with fewer visits, better care through more attention from health workers, and reduced travel costs were the primary reasons for preferring the integrative model.

Moreover, the study indicated that patients reporting anxiety/depression problems and history of non-medication-assisted drug rehabilitation, which is traditionally compulsory in Vietnam, were more likely to select integrative models. The former was known to be a predictor of drug relapse and HIV-related risk behaviors among drug users [42–44]. Meanwhile, the latter tended to seek for MMT and more willing to pay more for MMT than people without rehabilitation [45, 21]. Both subjects require the comprehensive health care services, which is potential to the integrative models.

It is noteworthy to observe that patients perceiving stigma in community were less likely to prefer for integrative model. In Asian culture, illicit drug use is considered “social evil,” and users frequently experience the isolation and rejection by their communities and families [46, 47]. Integrative models provide services for both drug users and others may result in the loss of privacy. Privacy is also the major reason to choose the stand-alone model among respondents. However, the proportion of MMT patient reporting discrimination was low in both groups. Furthermore, this study reveals that patients ever disclosing their drug addiction and related-health problems to other people were likely to prefer integrative model, emphasizes the importance of communication between drug users and their community [45]. Thus, addressing drug use-related stigma in community and facilitating drug users’ reintegration into society are essential factors that should be addressed in implementing integrative models.

### Preference for decentralized models

In the context of limited-resources settings, it is difficult to provide comprehensive, centralized services for substance abuse patients due to operational costs [48]. Thus, decentralizing by integrating into primary health care facilities has been emerging as a potential model for scaling up substance dependence treatment services [49, 37]. As decentralized HIV-related services, this model for opioid dependence treatment has benefits in reducing geographical barriers and cost of travel for patients and decreasing burden of care in high volume clinics [37, 21]. Notably, due to the lack of MMT clinics, some of MMT patients have to participate in available clinics that are far from their local areas, and these are not selected by their preference. Therefore, decentralized model will benefit them to access MMT easily. Consistent with previous investigations, distance from home was the main reason that decentralized MMT services at commune level were preferred.

Stigma and discrimination for illicit drug users at community are the obstacles for implementing decentralized model [37]. Despite lack of evidence about stigma in decentralized for MMT patients, a survey in South Africa indicated that HIV-positive people preferred to avoid the clinics in their locals and were willing to travel a long distance to hide their HIV status [50]. Our survey indicated that the main reasons to not select decentralized model was also to keep privacy. In Vietnam, illicit drug users experience stigma in community similarly with people living with HIV (PLWH) [51]. The result of multivariate regression did not find any relations between stigma and the preference for decentralized model; however, this factor is a concern for decentralization.

Notably, people participating in MMT services with comprehensive HIV services, as well as people having long duration of MMT, were less likely to prefer decentralized models. This phenomenon might explain by the poor quality of care in commune health centers compared to what they received in current facilities [52]. Moreover, the reason of better quality of care was reported by a small proportion of MMT patients, suggesting patients did not believe in the capacity of medical staff in primary care facilities. This result was fit with prior studies of decentralized ART program, revealing that the shortage of health staffs and trained providers should be considered if implementing this model [53–55].

### Study implications

The findings of this study suggested some implications to implement different MMT service models in Vietnam. First, since the prevalence of mental disorders was high among drug user population, MMT clinics should be integrated with general health and psychological health care services to provide timely comprehensive care to the patients. Second, when implementing integrated and decentralized MMT models, stigma and discrimination should be tackled by some strategies such as separating the areas for MMT-related services and general health care services, ensuring the confidential of MMT patients and assigning drugs for patients without easy identification [56]. In addition, health staffs should be equipped to support patients to cope with their social problems. Finally, sufficient trained health workers, convenient procedure, timely support, and adequate monitoring in primary health care facilities should be ensured to fulfill the criteria for performing integrative and decentralized model successfully [36, 57].

### Strengths and limitations

This is the initial study investigating the preference of MMT patients for different service delivery models. The findings will have significant contribution to make a policy that maximizes the efficiency of MMT in the context of limited-resources setting as Vietnam. Nonetheless, several limitations need to be considered. First, the data was based on self-reports, which may lead to recall bias. Combining other clinical indicators is essential to have validated measures. Second, the causal relations between MMT delivery models and the change of health status, health-related quality of life, and drug use pattern could not be investigated due to the cross-sectional design. Finally, convenience sampling technique may limit the generalization of study.

## Conclusion

In conclusion, the study confirmed high preference of MMT patients for the integrated and decentralized MMT service delivery models. The convenience of health care services utilization and reduction of geographical barriers were the main reasons to use those models among drug use population in Vietnam. The results also suggested the necessity of integrating not only general health care but also psychological supports with MMT services to provide comprehensive care for drug users. However, to implement this model, countering community stigma and encouraging the communication between patients and their societies needed to be concern in planning process. Those patients who had severe health and behavioral issues should still be managed at higher level while receiving methadone at local commune health station once their status is stable.
